# Nurses’ Motivations, Barriers, and Facilitators to Engage in a Peer Review Process: A Qualitative Study Protocol

**DOI:** 10.3390/nursrep13010029

**Published:** 2023-02-22

**Authors:** Júlio Belo Fernandes, Josefa Domingos, John Dean, Sónia Fernandes, Rogério Ferreira, Cristina Lavareda Baixinho, Cidália Castro, Aida Simões, Catarina Bernardes, Ana Silva Almeida, Sónia Loureiro, Noélia Ferreira, Isabel Santos, Catarina Godinho

**Affiliations:** 1 Grupo de Patologia Médica, Nutrição e Exercício Clínico (PaMNEC), 2829-511 Almada, Portugal; 2 Centro de Investigação Interdisciplinar Egas Moniz (CiiEM), 2829-511 Almada, Portugal; 3 Escola Superior de Saúde Egas Moniz, 2829-511 Almada, Portugal; 4 Triad Solutions, Aurora, CO 80012, USA; 5 Instituto Politécnico de Beja, Escola Superior de Saúde, Departamento de Saúde, 7800-111 Beja, Portugal; 6 Comprehensive Health Research Center, 7004-516 Évora, Portugal; 7 Nursing School of Lisbon, 1900-160 Lisbon, Portugal; 8 Nursing Research, Innovation and Development Centre of Lisbon (CIDNUR), 1900-160 Lisbon, Portugal; 9 Department of Nursing, Centro Hospitalar de Setúbal E.P.E., 2910-446 Setúbal, Portugal; 10 Department of Nursing, Hospital Garcia de Orta E.P.E., 2805-267 Almada, Portugal; 11 Department of Nursing, Unidade de Cuidados na Comunidade de Palmela, 2950-483 Palmela, Portugal

**Keywords:** nurses, peer review, motivation, challenges, difficulties, facilitators

## Abstract

Peer review supports the integrity and quality of scientific publishing. However, although it is a fundamental part of the publishing process, peer review can also be challenging for reviewers, editors, and other stakeholders. The present study aims to explore the nurses’ motivations, barriers, and facilitators in engaging in a peer review process. This qualitative, descriptive exploratory study will be developed in partnerships with three research centers. Researchers followed the consolidated criteria for reporting qualitative research (COREQ) checklist to ensure the quality of this study protocol. According to the selection criteria, the purposive sampling will be used to recruit nurse researchers that act as peer reviewers for several scientific journals in various fields of knowledge. Interviews will be conducted until data have been sufficiently consistent with meeting the initial objectives. Researchers will develop a guide comprising a set of open-ended questions to collect participants’ characteristics, descriptive review behavior, and perceptions regarding their motivations, barriers, and facilitators. Researchers will analyze data using an inductive process of content analysis with the help of the QDA Miner Lite database. Findings from this study will generate knowledge that may help stakeholders identify facilitating factors and barriers and guide the development of strategies to remove or minimize these barriers.

## 1. Introduction

Since the 17th century, peer review has been vital in scientific communication in journals [1]. The process of peer review advanced more systematically as a result of the measurable development of research output and expanded fields of specialization [2,3]. Although the concept of peer review is ordinarily used, we can identify different definitions of the term in various scientific journals [3]. Regardless of the several kinds of peer review, the focal aim is to guarantee the integrity and quality of research by evaluating the articles’ quality, validity, and originality [4]. The peer reviewer role implies assessing the submitted manuscripts and providing truthful and unbiased feedback to both the authors and editors. To act as peer reviewers, nurses, along with being fair, unbiased, and professional, must be experts in the research being reviewed [5]. By assuming the reviewer role, nurses agree to protect the vital principles of scientific research and to safeguard that research in a practiced manner that is best and safest for the study participants. Peer review is at the heart of the practices of not just scientific journals but of all of science [6]. It supports the integrity and quality of all scientific publishing processes [5]. When employed correctly, peer review has the potential to move science forward [7].

Although it is a crucial part of the scientific publishing process, peer review can also be a largely unrecognized and unacknowledged role. The peer review process can be unquestionably challenging from different perspectives. Authors must face criticism and respond by defending or changing their points of view. Editors sometimes must make tough decisions on their fellow scientists’ work, and peer reviewers spend their time uncompensated [8]. Publons estimated that in 2018–2019 the prime 1% of peer reviewers of scientific papers had invested more than 1.3 million hours reviewing [5].

Peer reviewing should be a rewarding task that not only enhances the trust and understanding in research but also has the potential to hasten the nurse’s research and academic career [5,9].

The peer review process is one of the most authentic cases of how fellow nurse scientists support one another in shaping a field. All processes of peer review, including the editors’ guidance, reviewers’ comments, and the authors’ responses to debating or acknowledging the expert opinions can give rise to insights of all kinds and inspiration [8].

One major step of the peer review process is choosing adequate peer reviewers. This step is a gatekeeper to the entire peer review process, and although this might appear to be an easy task, this step can be very challenging and rate-limiting for editors. Unfortunately, it is common for a manuscript’s assigned editor to obtain recurrent peer rejections to review an article. Even though nurses might have legitimate reasons for refusal, this decision makes the peer review process challenging and time-consuming [10].

As the role of peer reviewer receives more recognition, we expect it will encourage more fellow nurse researchers to accept this role. However, at present, the quest to find committed, high-quality, experienced nurse reviewers available to invest their time in the review process is the core challenge for scientific journals worldwide [5]. This quest becomes even more challenging in nursing because there are various fields in which a nurse may decide to specialize. The guide to nursing specializations and concentrations lists more than 100 nursing specialties [11]. This varied offer allows nurses to advance towards acquiring knowledge and skills in different fields, becoming experts in that area. However, this high level of specialization may limit their participation in peer review processes related to topics outside their specific area of expertise. In addition, we should consider the worldwide shortage of nurses [12], and the results of several studies demonstrating the burden felt by these professionals [13,14,15].

There is an unmet need to understand these challenges and how to overcome them and facilitate the review process, and to offer more support to reviewers. There is limited knowledge about the reviewers’ perspective on participating in the peer review process; therefore, this research explores the nurses’ motivations, barriers, and facilitators to engaging in a peer review process.

## 2. Methods

### 2.1. Study Design

This is a qualitative study that will use a descriptive exploratory design to explore the nurses’ motivations, barriers, and facilitators to engaging in a peer review process. Descriptive exploratory designs are used to investigate a research phenomenon that has not been studied in depth and gather a deeper understanding of a specific phenomenon and its context [16,17].

To ensure the quality of this study protocol, researchers followed the consolidated criteria for reporting qualitative research (COREQ) checklist [18].

### 2.2. Time Period

The study will be developed from January 2023 to December 2023 (Gantt Chart, Table 1).

### 2.3. Setting and Participants

The research sample of this study will include scientific journal reviewers holding academic titles in nursing sciences from three research centers in Portugal. We will focus more on the richness of the cases selected than on the sample size [19,20].

We will reach out to the following:

- The Nursing Research, Innovation, and Development Centre of Lisbon (CIDNUR) is a differentiated unit of the School of Nursing in Lisbon dedicated to developing research. The CIDNUR’s mission is to develop fundamental, applied, and experimental research in nursing in line with the Sustainable Development Goals and the principles of Open Science.
- The Research Centre of Egas Moniz—Cooperativa de Ensino Superior CRL (CiiEM) represents a nucleus of innovation and knowledge creation. It fosters a paradigm of translational research and teaching in collaboration with other functional structures of Egas Moniz. It also focuses strongly on community interactions in different contexts, including health, health-related sciences, and social services.
- The Comprehensive Health Research Center is a center of excellence for research, training, and innovation in health promotion, prevention, rehabilitation, and healthcare services. This consortium comprises healthcare professionals, researchers, academics, patients, and entrepreneurs who work together toward a common goal. This research center provides a unifying environment for health research, innovation, and education in public health, lifestyle, nursing, rehabilitation, and clinical research.

In 2022, these research centers aggregated 590 researchers that act as peer reviewers for several scientific journals in various fields of knowledge.

The inclusion and exclusion criteria were as follows.

Inclusion criteria:

- Be a member of one of the research centers;
- Hold an academic title in nursing sciences;
- Had performed at least one peer review for a scientific journal;
- Willingness to participate in the study.

Exclusion criteria:

- Target population unwillingness to participate or comply with all the proceedings.

Researchers will use purposive sampling, widely used in qualitative research, to identify and explore data related to the object of interest. This sampling method will allow the researcher to collect data from the best-fit participants and ensure that the results are relevant to the research context [21,22].

For the first phase of the sample selection, researchers will ask the research centers to provide an initial list of nurse professors/researchers who comply with the study inclusion/exclusion criteria. To obtain sample variation, in the second phase, we will use three criteria: academic title (Assistant Professor, Associate Professor, and Full Professor), time of experience, and time between participation in peer review processes. A previous study revealed differences between tenured and non-tenured referees in the time taken to review [23].

For this study, researchers will consider saturation, as proposed by Glaser and Strauss [24], where researchers will continue to interview new participants until data have reached sufficient consistency to meet the initial objectives. The criteria to decide when to stop sampling the different groups pertinent to a category is when data about a construct reveal no new properties nor yield any further theoretical insights regarding the phenomenon of study whereby researchers can develop properties of each category.

### 2.4. Data Collection Procedures

The leading researcher will be responsible for recruiting eligible participants and collecting their written informed consent. Participants will be contacted via telephone or email, presenting the research project, its aims, and the importance of the participant’s collaboration.

Researchers will safeguard that the chosen location will be free of noise in an environment that ensures the participants’ privacy and comfort. Therefore, interviews will be conducted in the office of the leading researcher or the participant’s office. No one else will be present during the interviews besides the participant and the interviewer. Every interview will be audio-recorded.

The interviews will be conducted by an experienced researcher: a skilled interviewer [25,26,27] with a Ph.D. in Nursing Sciences who has no prior relationship with the participants.

The semi-structured interview will follow an interview guide to ensure that central issues are addressed. Reviewers will develop a guide comprising a set of open-ended questions to collect participants’ characteristics and descriptive review behavior, covering the following:

- the reviewers’ characteristics (sex, age, time of professional experience, time of experience as reviewer), and academic title;
- the characteristics of the participant’s behavior (research productivity—measured by the number of articles in Scopus or Web of Science; the quantity of declined reviews; the average number of manuscripts reviewed per year; and the average time invested in each review).

A second set of questions will be developed to gather participants’ perceptions regarding the following:

- the motivation for conducting reviews and the outcomes perceived;
- the reasons for declining to review;
- the barriers for review;
- the costs associated with conducting reviews;
- the reasons for accepting reviews;
- the facilitators for review;
- the incentives for the review;
- the relation between the review and the job description.

The guide will be pilot tested among colleagues to assess its suitability for this study. Colleagues will be questioned to know their perceptions regarding the interview guide and determine if they consider it to be sufficiently clear, objective, comprehensive, and to not present questions that could be ambiguous or equivocal.

Based on previous experience, researchers estimate that each interview will take approximately 40 min.

### 2.5. Data Analysis

We will transcribe verbatim the audio-recorded interviews into a Word file. The final version of the interview transcripts will be returned to participants to assess any discrepancies and provide additional elucidation that may improve data accuracy.

To guarantee the participants’ anonymity, in the verbatim transcription, the participants’ identification will be replaced by a unique code number (for example, P1, P2, P3, etc.).

Descriptive statistic measures of count, mean (variables sex and academic title), standard deviation, median, minimum, maximum, and range (variables age, time of professional experience, and time of experience as reviewer) will be computed for sample characterization, using the IBM Statistic Package for the Social Sciences software (IBM Corp. Released 2020. IBM SPSS Statistics for Windows, Version 27.0. Armonk, NY, USA: IBM Corp.).

The data analysis of open-ended questions will be conducted by two researchers independently. Textual data from open-ended questions will be exported to the QDA Miner Lite database and analyzed using an inductive process of content analysis as described by Braun, Clarke, Hayfield, and Terry [28]. This process will involve comprehensive readings of the interview transcripts to derive concepts and categories, which allow findings to arise straight from the data analysis rather than from a priori expectations or models.

Therefore, researchers will start with reading the interviews in detail several times. This process will identify differences and similarities between the participants’ speech, allowing researchers to see patterns and create initial categories.

The text will be separated into different meaning units of words, phrases, and passages that focus on the same topic. Then, researchers will assign codes to the meaning units and categories using the participants’ own words. The codes will reflect the differences and similarities in the participants’ perceptions regarding the phenomenon in the study.

Any differences identified during the analysis will be resolved by discussion between the two researchers. If a consensus is not reached, a third researcher will analyze the discrepancy.

Afterward, the research team will review the analysis of data made by the first two researchers and match each quote to one of the identified themes. Finally, the categories and organizing framework will be shared with other researchers external to the study to ratify the final results.

### 2.6. Trustworthiness

We will implement practices recommended by Nowell, Norris, White, and Moules [29] to ensure the study’s trustworthiness. To guarantee credibility, researchers will discuss and detail every decision until consensus during the analysis process and return the initial themes and organizing framework to participants to ratify the researchers’ interpretations. In addition, the information on the participants’ characteristics and study context will be provided in detail, and participant quotations will be provided through the study report to ensure that readers who sought to transfer the findings to another context could judge transferability. Regarding the study’s dependability, researchers will detail every step of the decision-making process to ensure readers can follow the research. Finally, to safeguard confirmability, researchers will request a team of external investigators experienced in qualitative research to search for inconsistencies by comparing their perceptions with those of the researchers.

### 2.7. Ethics and Procedures

Researchers will conduct this research following the Helsinki Declaration (as revised in 2013) and seek approval from the research center’s ethics committee.

In addition, all the participants must sign the informed consent form. The informed consent form will state that participation in this study is entirely voluntary. Therefore, participants are free to not reply to some questions, change or review their responses, or voluntarily quit at any time.

All data will be conducted in compliance with ethical principles guaranteeing the participants’ anonymity and confidentiality. Consequently, no individual data will be accessible, a unique code number will replace participants’ identification, and only the leading researcher and the interviewer will have access to the identification sheet.

The leading researcher will archive vital documents in a way that ensures that they are readily available, upon request, to the competent authorities. All paper documents will be stored in a locked file. Digital data will be coded and stored on a password-protected computer. After the verbatim transcription, all the audio-recorded data will be destroyed. All data will remain locked in a file cabinet at Egas Moniz University for five years. The leading researcher will destroy all data when this retention period is complete.

## 3. Discussion

Little is known about the factors that influence the review process. We will study the nurses’ motivations, barriers, and facilitators to engage in a peer review process using qualitative research. This study will improve our knowledge regarding the challenges reviewers face in participating in a peer review process and what motivates them to continue to perform peer reviews. Identifying these factors may allow the development of strategies to support nurses’ participation in a peer review.

Through peer review, scientific journals validate the published research manuscript and automatically receive credibility [1,5]. High-quality peer review is significant to legitimize the science we publish [5]. A fair, unbiased, and quality review can be challenging to achieve [6]. Although peer review is considered vital to academic quality [4,5] and has the potential to move nursing science forward [7], it is challenging for editors for several reasons, such as difficulty in recruiting expert reviewers with a variety of areas of knowledge and bias in choosing from a list of reviewers who might not be willing or have time available to review the article over the established period [4,6,30].

Even in the case of rejection, when peer preview is performed correctly, it can have positive results for nursing science, as editors will communicate that decision followed by an explanation of the manuscript limitations that led to its rejection and the reviewers’ constructive suggestions on how the research could be improved [5].

After completing this study, the team of researchers will work with the research centers to address the barriers to engaging in a peer review process. The peer review process is at the heart of the practices of all science [6]. It is imperative that research centers, in addition to scientific journals, develop strategies that support the participation of nurse researchers in peer review processes.

We draw attention to the fact that peer review needs to be a highly acknowledged and recognized role by the scientific community. The discussion concerning the consequences of external incentives for peer reviewers remains open. Previous studies analyzing the effects of the external review incentives given by the journal or affiliated institution were not enlightening, as Zaharie and Osoian’s [3] study identified that the reviewers’ internal motivation diminishes in the presence of external rewards. Unlike these results, Chetty, Emmanuel, and Laszlo´s study [23] revealed that cash incentives significantly improve the speed of the reviews. Social incentives have smaller but significant effects on review times and are especially effective among tenured professors, who are less sensitive to deadlines and cash incentives. However, the incentives provided have little or no effect on engagement rates in peer review, quality of reports, or review times at other journals.

We also draw attention to the fact that the publication of study protocols can enhance research transparency, decrease publication bias, prevent duplication of research, and alert the scientific community to know what trials are planned or ongoing, enabling other researchers to adapt and build upon previous researchers’ accomplishments [31]. In addition, expert peer review feedback has the potential to help to refine and shape the submitted protocol [32]. Therefore, the publication of study protocols can help improve the medical research standard. We can identify many published study protocols in the literature, from quantitative [33,34,35] to qualitative [36,37,38] investigations to literature review protocols [39,40,41]. As in previously published study protocols, with the writing of this study protocol, we intend to present the aims, methodological approach, and plan to operationalize the research. The results are expected to have straight relevance to the scientific community.

We recognize that the study has limitations. First, researchers and scholars with strong views toward peer review may be unwilling to participate in this study due to perceptions related to social desirability resulting in a possible bias in participant recruitment. Alternatively, if participants perceive it to be socially desirable, they might overstate the frequency of their behavior, increasing the frequency of the positive items. Second, as in preceding studies that rely on data collected from interviews, the participants’ perceptions and feelings might deviate from what they expose due to biases such as a lack of confidence in guaranteeing anonymity or protecting identity, values, or beliefs. To overcome these possible limitations, we rely on the research team’s skills and experience to ensure that participants trust that their personal information, values, and beliefs will be kept anonymous and confidential while providing rich and detailed accounts of their perceptions regarding the motivations, barriers, and facilitators to engaging in peer review.

## 4. Conclusions

This study will be the first to explore the nurses’ motivations, barriers, and facilitators to engaging in peer review. The study findings can have practical implications for stakeholders to enable improvements in the peer review process. By developing strategies to remove or minimize the influence of the identified barriers and focus on the factor that facilitates the engagement in the peer review process, stakeholders can enable fellow nurse scientists to engage in high-quality peer reviews.

## Figures and Tables

**Table 1:** Project schedule.

Months	1	2	3	4	5	6	7	8	9	10	11	12
Planning	x	x	-	-	-	-	-	-	-	-	-	-
Ethical approval	-	-	x	x	-	-	-	-	-	-	-	-
Participant recruitment	-	-	-	-	x	x	-	-	-	-	-	-
Data collection	-	-	-	-	x	x	x	-	-	-	-	-
Data analysis	-	-	-	-	-	x	x	x	x	x	-	-
Reporting	-	-	-	-	-	-	-	-	-	x	x	x

x: Execution time.

## Data Availability

Not applicable.
